# DNAemia Detection by Multiplex PCR and Biomarkers for Infection in Systemic Inflammatory Response Syndrome Patients

**DOI:** 10.1371/journal.pone.0038916

**Published:** 2012-06-15

**Authors:** Catherine Fitting, Marianna Parlato, Minou Adib-Conquy, Nathalie Memain, François Philippart, Benoît Misset, Mehran Monchi, Jean-Marc Cavaillon, Christophe Adrie

**Affiliations:** 1 Unit Cytokines & Inflammation, Department of Infection and Epidemiology, Institut Pasteur, Paris, France; 2 Service de reanimation, Delafontaine Hospital, Saint Denis, France; 3 Service de réanimation médico-chirurgicale, Groupe hospitalier Paris Saint-Joseph, Université Paris Descartes, Paris, France; 4 Service de Soins Intensifs, Jacques Cartier Hospital, Massy-Palaiseau, France; 5 Service de Physiologie et d’exploration fonctionnelles, Cochin Hospital, University of Paris Descartes, Sorbonne cite, Paris, France; University of São Paulo, Brazil

## Abstract

Fast and reliable assays to precisely define the nature of the infectious agents causing sepsis are eagerly anticipated. New molecular biology techniques are now available to define the presence of bacterial or fungal DNA within the bloodstream of sepsis patients. We have used a new technique (VYOO®) that allows the enrichment of microbial DNA before a multiplex polymerase chain reaction (PCR) for pathogen detection provided by SIRS-Lab (Jena, Germany). We analyzed 72 sepsis patients and 14 non-infectious systemic inflammatory response syndrome (SIRS) patients. Among the sepsis patients, 20 had a positive blood culture and 35 had a positive microbiology in other biological samples. Of these, 51.4% were positive using the VYOO® test. Among the sepsis patients with a negative microbiology and the non-infectious SIRS, 29.4% and 14.2% were positive with the VYOO® test, respectively. The concordance in bacterial identification between microbiology and the VYOO® test was 46.2%. This study demonstrates that these new technologies offer great hopes, but improvements are still needed.

## Introduction

Sepsis is a common cause of morbidity and death in intensive care units [1]–[3]. The diagnosis of sepsis is difficult, because clinical signs of sepsis often overlap with other non-infectious causes of systemic inflammation. These signs include tachycardia, leukocytosis, tachypnea, and pyrexia, which are collectively termed a systemic inflammatory response syndrome (SIRS). SIRS is very common in critically ill patients, being found in various conditions including trauma, surgery, burns, pancreatitis, post-cardiac arrest syndrome, cardiac surgery (particularly those requiring extracorporeal circulation) and hypoxic injuries [4]–[6]. This means these signs can be misleading as critically ill patients often display a SIRS without infection [7]–[10]. Microbiological culture can be used to distinguish sepsis from non-infectious conditions. However, blood culture lacks sensitivity, and there is often a substantial time delay. This issue is of paramount importance, since therapy and outcome differ greatly between SIRS patients with or without infection. Clinicians are often prone to overuse antibiotic therapy, for fear of not treating a potential infection or superinfection, however, the widespread use of antibiotics for all such patients is likely to increase antibiotic resistance, toxicity, and costs [11]. On the other hand, any delay in administration of antibiotics can be extremely detrimental for a severely ill infected patient with an exponential increase in the odds ratio for death [12], [13]. The search for early biomarker tools for the diagnosis of infection, such as procalcitonin (PCT) and soluble Triggering receptor expressed on myeloid cells-1 (sTREM-1), initially yielded promising results in discriminating sepsis from aseptic SIRS. However, these results have been widely challenged and are now considered controversial because they seem more related to the inflammatory response, irrespective of the cause [14], [15]. Furthermore up to 40% of the infections remain strongly suspected but not bacteriologically documented [3], [16].

Research is ongoing to find new markers, profiles or combinations of markers to better discriminate SIRS related to infection from SIRS unrelated to infection. Many biomarkers have been under scrutiny such as copeptin [17], proendothelin-1 [18], proadrenomedullin [19] identified by proteome analysis. Cytokine profiles using multiplex analysis seem more related to the severity of the SIRS than the trigger of the SIRS (infectious or non-infectious diseases) [20]. Thus, new tools have been developed to identify bacteria by detecting their DNA using various techniques such as hybridization probe assays and real time polymerase chain reaction (PCR). These techniques have many potential interests over conventional microbiological tests including: decreased delivery time (within approximately 2–8 hours), reduced risk of negative culture due to prior use of antibiotics, detection of slow or fastidiously growing organisms and discrimination between non-infectious and infectious SIRS, thus decreasing the use of antibiotics. However, these tests remain to be validated in a clinical setting.

The goal of the current study was to evaluate the diagnostic value of a number of biomarkers that may help to distinguish infectious from non-infectious SIRS and possibly identify the causative pathogen by plasma detection of microbial DNA, in intensive care unit (ICU) patients with a (clinically) suspected bacterial infection.

## Results

### Patients

We planned to include a first cohort of patients and then if the results had been highly positive to include 300 more patients as a confirmatory cohort. Based on the results reported in this first cohort, we decided not to further pursue the study. We included 83 patients with sepsis and 18 with non-infectious SIRS. Due to insufficient amounts of DNA recovered after the Looxster® step, contamination or missing tubes, only 72 patients with sepsis and 14 non-infectious SIRS could be analyzed.

The majority of sepsis patients were male (62.55%). The median age of patients was 73 years {55–80} and median SAPS II score and SOFA score at admission were 50 {34–65} and 7 {5–11} respectively. Fifty seven percent of the patients required vasoactive or inotrope support at inclusion. The infection was located in the lungs (53%), abdomen (23%), urinary tract (14%), or other sites and multi-site (13%). The infection was community-acquired for 60% cases, hospital-acquired for 27% cases and ICU-acquired for 13% cases. Seventeen sepsis patients had no positive microbiological samples. ICU mortality was 29.7% among sepsis patients and 28.5% among SIRS patients (table 1).

**Table 1:** Patients’ characteristics and dosages (median {range}).

Variables	Sepsis (n = 72)	Non-infectious SIRS (n = 14)	p
**At admission**			
Age (years)	73 {55–80}	70 {67–83}	0.32
Male (%)	45 (62.5%}	9 (64%)	0.89
SAPSII,	50 {34–65}	46 {42–65}	0.70
SOFA score	7 {5–11}	8.5 {5–9}	0.54
**At inclusion**			
SOFA Score	7 {5–11}	7 {4–11}	0.59
White blood cells (10^9^/L)	15 {11–23}	14 {12–19}	0.80
Hemoglobin (g/dl)	10.4 {9–12.9}	11.3 {10.6–11.6}	0.18
Platelets (10^9^/L)	216 {128–337}	119 {132–25}	0.87
Urea (mmol/L)	12.3 {7.1–19.1}	12 {8.6–13.5}	0.74
Creatinine (µmol/L)	114 {7.1–19.1}	152 {8.6–13.5}	0.06
Alanine transaminase (U/L)	34 {20–63}	34 {22–122}	0.54
Aspartate transaminase (U/L)	49 {27–71}	27 {21–130}	0.69
Norepinephrine and/or epinephrine (mg/h)	0 {0–2.8}	0 {0–2.1}	0.09
Positive balance (ml/day)	2875 {1365–4960}	1350 {500–2000}	0.01
ICU Mortality (%)	22 (30.5%)	4 (28.5%)	0.77
Lactate (mmole/L)	2 {1.5–3.3}	2.2 {1.8–3.4}	0.17
CRP (mg/L)	195 {110–286}	76 {22–110}	<10^–4^
Pro-Calcitonin (ng/mL)	4.84 {1.4–17.2}	0.4 {0.16–14}	0.06
IL-1Ra (pg/mL)	297 {114–1208}	776 {139–7272}	0.22
IL-6 (pg/mL)	151 {82–384}	224 {28–337}	0.81
IL-8 (pg/mL)	124 {53–268}	182 {28–275}	0.79
IL-10 (pg/mL)	11 {4–20}	19 {12–42}	0.07
MCP-1 (pg/mL)	104 {41–289}	125 {76–362}	0.40
TNF (pg/mL)	22 {14–32}	26 {19–51}	0.13
Peptidoglycan (pg/mL)	50 {0–65}	20 {0–90.6}	0.84

Amongst the non-infectious SIRS, 64% were male. The median age of patients was 70 years {67–83} and median SAPS II score and SOFA score at admission were 46 {42–65} and 8 {5–9} respectively. One patient had a successfully resuscitated cardiac arrest, 1 had hemorrhagic shock, 2 had severe cardiogenic shock requiring inotropes or vasoactive agents, and 10 were post-operative cardiac surgery patients requiring extracorporeal circulation (the latter requiring vasoactive support in 9 cases).

### Blood Cultures and VYOO® Analysis

**Figure 1:**
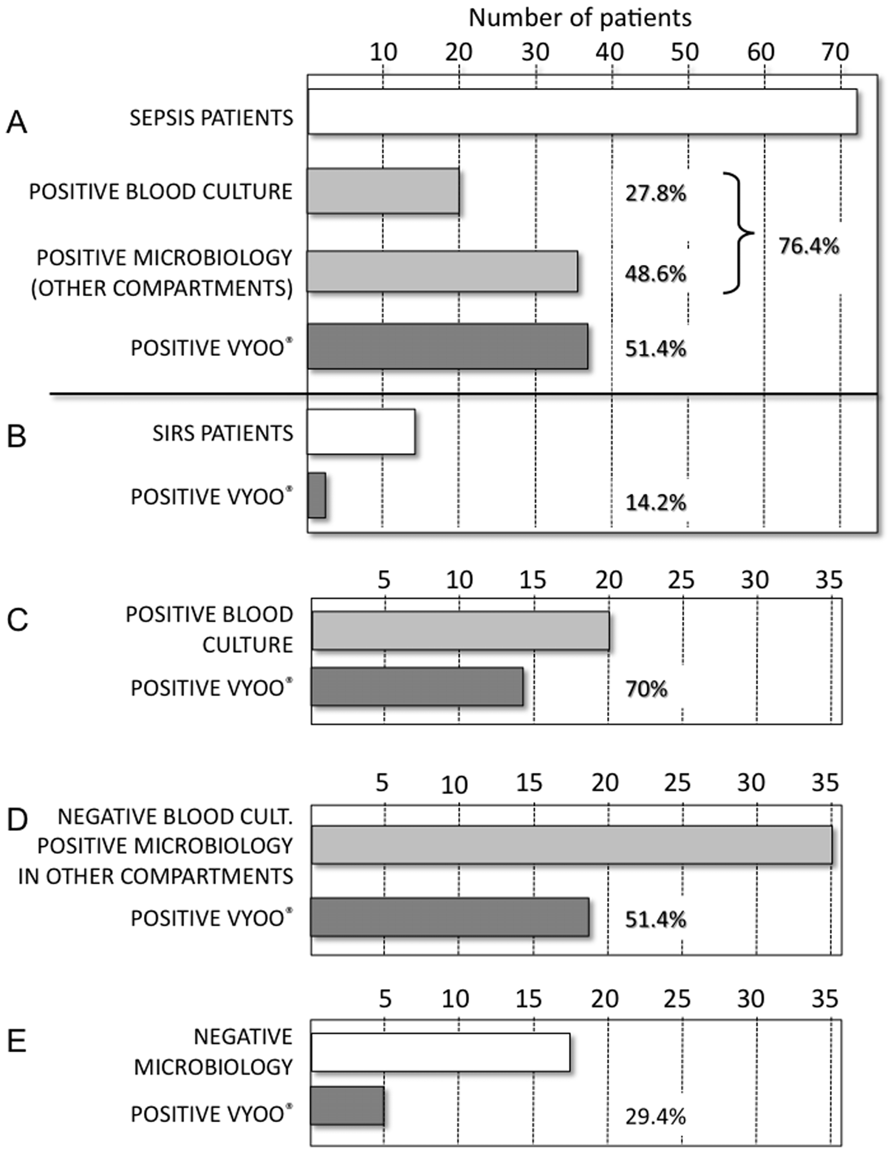
Microbiology analysis in sepsis and SIRS patients. A. Number of sepsis patients with positive blood culture, positive microbiology in other compartments (BAL, urine, catheter, ascitis, peritoneal fluid, synovial fluid, cerebrospinal fluid, bile, skin and bone biopsies), and total number of sepsis patients with a positive VYOO® test. B. Number of non-infectious SIRS patients with a positive VYOO® test. C. Number of sepsis patients with a positive blood culture who had a positive VYOO® test. D. Number of sepsis patients who had a negative blood culture but had a positive microbiology test in other compartments who had a positive VYOO® test. E. Number of sepsis patients without any positive microbiology test who had a positive VYOO® test.

Twenty sepsis patients had a positive blood culture and 35 had a positive culture in another biological sample (i.e. broncho-alveolar lavages, urine, catheter, ascitis, peritoneal fluid, synovial fluid, cerebrospinal fluid, bile, skin, or bone biopsies) (figure 1). According to blood cultures and cultures of other biological samples, infections were due to Gram-negative bacteria (14 cases), Gram-positive bacteria (20 cases), mixed Gram-negative and Gram-positive bacteria (12 cases), anaerobes (2 cases), and Candida spp (1 case). Five cases were displaying both fungal (4 *Candida albicans*, 1 *C. lusitaniae*) and bacterial infection in other compartments thanblood, and 18 had no identified organism.

**Figure 2:**
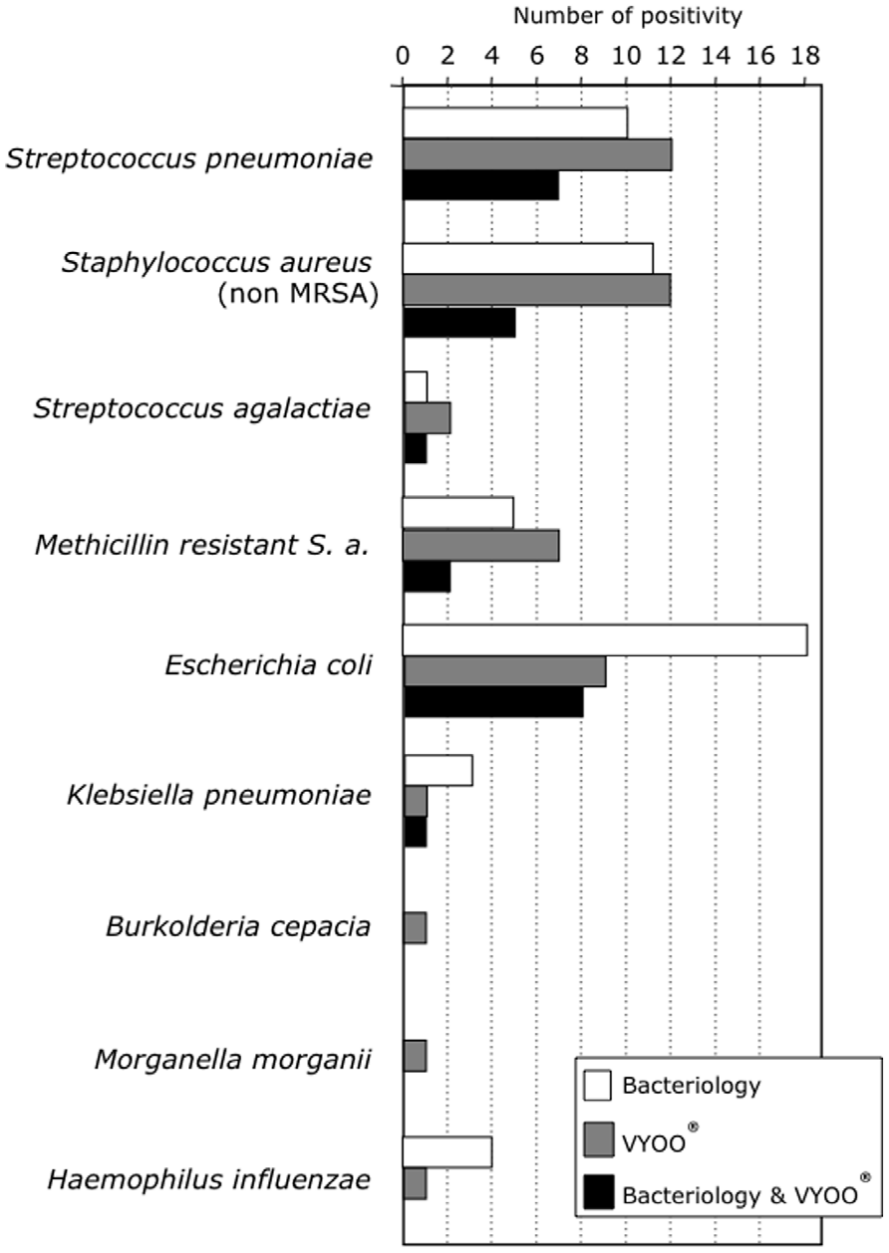
Matching between microbiology and bacterial DNA analysis. Comparison of the bacterial identification by classical microbiological analysis or by the VYOO® technology. White bars: identification by classical microbiology in any compartments; grey bars: identification by VYOO® test in blood samples; black bars: matching between microbiology and VYOO® test.

Microorganisms were detected in the blood of 37 out of 72 sepsis patients (51.4%) with the VYOO® technique and the results were better amongst those with a positive blood culture: 14/20 (70%) were accurately detected (table 2). Interestingly, 9 patients with identified organisms at the site of infection were correctly detected with the VYOO® technique without concomitant septicaemia. For two additional positive PCR in the septic cohort, the microorganism detected was compatible with non-documented sites of infection amongst these patients (Pneumonia/*Streptococcus pneumoniae* and severe colitis/*Morganella morganii*). Two positive VYOO® detections were obtained among the non-infectious SIRS cohort (14,2%), neither of which had a documented infection based on classical bacteriological tests. No fungal infections were detected using the VYOO® technology while microbiology revealed one positive blood culture *(C. albicans)* and positivity in five other samples (1 peritonitis, 3 lung infections, 1 surgical site). According to microbiology, 48% of the patients were infected with Gram-negative bacteria, among whom 28% were revealed by the VYOO® technology. Among the pathogens revealed by microbiological analysis and the VYOO® technology, *E. coli* was the most prominent Gram-negative bacteria (34,6% and 19,6% respectively) (figure 2). Among Gram-positive-bacteria the frequency of *Staphylococcus aureus* (incl. MRSA) was 22.7% according to microbiological analysis and 30.8% according to the VYOO® technology. The correspondence in bacterial identification between microbiology and the VYOO® technology was 46.2% (figure 2).

**Table 2:** Microbiology and VYOO**®** test in sepsis patients with positive blood culture.

	Positive Blood Culture	VYOO® test +
*Staphylococcus aureus*	2	3
Methicilin resistant *Staphylococcus aureus*	4	2
*Pseudomonas aeruginosa*	1	0
*Streptococcus pneumoniae*	3	3
*Escherichia coli*	6	4
*Klebsiella pneumoniae*	1	1
*Enterobater chloacae* + *Acinetobacter baumanii* (2 organisms in the same blood culture)	1	0
*Neisseria meningitidis*	1	0
*Streptococcus oralis* + *Candida albicans* (2 organisms in the same blood culture)	1	0
*Haemophilus influenzae*	0	1
**TOTAL**	**20**	**14**

### Clinical and Biological Parameters

Univariate analysis showed that amongst the clinical and biological parameters, only fluid balance and CRP were significantly higher in sepsis. CRP was statistically significant after multivariate analysis (OR: 2.78 {IC95%: 1.005–1.029}, p<10^–4^). All other variables did not discriminate between the two groups, including inflammatory cytokines (TNF, IL-6, IL-8, MCP-1 (CCL2)), anti-inflammatory cytokines (IL-10 and IL-1 Ra), and circulating peptidoglycan (Table 1), and there was a trend of higher levels of pro-calcitonin (p = 0.06) and IL-10 (p = 0.07) (Table 1).

## Discussion

A diverse array of new experimental approaches are now available for the detection and identification of bloodstream infections, such as matrix-assisted laser desorption ionization time-of-flight mass spectrometry (MALDI-TOF MS) [21], [22], or pyrosequencing [23], [24]. The most studied approach is multiplex real-time PCR [25], [26], [27]. Despite the fact that this has been reported as a promising technology, validations are still required [28]. Among those systems already commercially available, Septifast® has been the most studied [29], [30], [31], [32], [33], [34], however results have been inconsistent (for review see [35]).

In our study, we used another available technique for multiplex PCR that detects a predefined panel of the most important sepsis pathogens by electrophoretic separation of target-specific amplicons (VYOO®, SIRS lab, Jena, Germany) associated with a specific enrichment of bacterial DNA. Only two meeting abstracts [36], [37] have been published, both in 2009, on bacterial and fungal detection with this particular approach. Here we report the first study using this approach in sepsis and non-infectious SIRS patient. In the cohort of patients with an infection, 70% of patients with positive blood cultures also gave a positive result using the VYOO® technique. Interestingly, we also found a good concordance between the PCR detection and the bacteriological results observed at the site of infection in nine cases without bacteremia. Furthermore, we found two PCR results to be compatible with pathogens usually seen at the site of infection responsible for the sepsis in patients without a documented bacterial infection. Overall these results seem to be comparable to those published using Septifast® [29], [38]. Furthermore, we did not find any data in favor of bacterial translocation in non-infectious SIRS, as may have been expected, particularly in the post cardiac surgery subgroup. We were also unable to detect *Candida* as described with this technique [37], and this is in accordance with a previous study using Septifast® [32], even in cancer patients who are heavily colonized by this pathogen [38]. The limited capacity to detect fungal DNA may be due either to some technical problems linked to the nature of the infectious agents, the low presence of fungi within the blood stream, or the low levels of fungal circulating DNA when fungi are present within the tissues. Of note, we had very few fungal infections in our cohort.

We also measured a number of clinical and biological parameters as well as various markers and cytokines. Interestingly, the severity or organ dysfunction scores were similar in both groups and, after multivariate analysis only CRP was able to discriminate infectious from non-infectious SIRS. We have previously shown that PCT is not associated with infection after correction for shock severity (fluid balance or levels of catecholamines’ administration). Similarly to what we have already observed with endotoxins, we studied here the circulating peptidoglycan and again did not find any difference between infectious and non-infectious SIRS.

In conclusion, the VYOO® approach for detecting microbial DNA is technically demanding, requiring around 8 hours of work. Despite encouraging results, its accuracy needs to be improved as we found a significant number of false positive results. Furthermore, some patients may be infected by several pathogens and it would be dangerous not to consider those that are not detected by the VYOO® test. A limitation of our study was a rather low number of patients. Because of our results, in accordance with our statistician, we decided not to perform the scheduled validation cohort because there was a limited chance to prove something else. A larger study would be required after improvement of the test. We are at the beginning of new era of molecular diagnostics in septic patients; the results are encouraging but their current accuracy needs to be improved before they can be considered a reliable tool for clinicians and patient care.

## Materials and Methods

### Study Protocol

The protocol has been approved by the ethical committee of Pitié-Salpétrière Hospital, Paris (NCT00698919), and written informed consent was obtained from either the patient or the next of kin. All ICU patients older than 18 years old, with a SIRS, severe sepsis or septic shock were included in this cohort study. SIRS, Severe sepsis and septic shock were defined according to the definition used by a panel of experts from the American College of Chest Physicians/Society of Critical Care Medicine [16], [39]. Three medico-surgical ICUs participated in this study (Delafontaine Hospital, 17 beds; Jacques Cartier Institute, 24 beds; and Saint-Joseph Hospital, 12 beds).

All codes and definitions were established prior to study initiation. The following information was recorded: age, sex, admission category (medical, scheduled surgery, or unscheduled surgery), origin (home, ward, or emergency room), McCabe score [40] and ICU and hospital mortality. Illness severity was evaluated on the first day in ICU using the Simplified Acute Physiology Score (SAPS II) [41], and Sepsis-related Organ Failure Assessment (SOFA) score [42]. Knaus scale definitions were used to record preexisting chronic organ failures including respiratory, cardiac, hepatic, renal, and immune system failure [43]. The presence or absence of infection was documented according to the recently updated standard definitions developed by the Centers for Disease Control [44]. In addition, quantitative cultures of specimens obtained by bronchoalveolar lavage, protected specimen brush, protected plugged catheter [45], or tracheal aspiration were required to diagnose ventilator-associated pneumonia. Community-acquired infection was defined as infection manifesting before or within 48 hours after hospital admission. Hospital-acquired infection was infection manifesting at least 48 hours after hospital admission but before ICU admission. ICU-acquired infection was diagnosed at least 48 hours after ICU admission. We observed in our database that 20 species represented more than 90% of the infection, so we grouped the most important pathogens depending of the place of acquisition because the others were too rare to be specifically studied. Infection sites were categorized as follows: pneumonia, peritonitis, urinary tract infection, exacerbation of chronic obstructive pulmonary disease, catheter-related infection, primary bacteremia (excluding untreated *Staphylococcus epidermidis* bacteremia), miscellaneous sites (mediastinitis, prostatitis, osteomyelitis, and others), and multiple sites. Early appropriate antimicrobial therapy was defined as effectiveness on the causative agent of at least one of the empirically selected antimicrobials on the day of the diagnosis of an episode of severe sepsis. Effectiveness of antimicrobials was assessed based on the culture results and known susceptibility of the organism to the antimicrobials used and on antimicrobial susceptibility testing. For nonfermenting Gram-negative bacilli, aminoglycoside monotherapy was considered inappropriate [46].

We determined three groups: 1) Patients with documented infection 2) Patients without infection, and 3) Patients with suspected infection but no documented bacterial infection. The type and number of microbiological samples taken was at the discretion of the clinician in charge of the patient. The presence or absence of infection, the sites of infection and causative pathogens, were determined by the medical staff of each unit and crossed checked by external validation (CA, FP, MM). Most of the VYOO® tests have been done on the first day of the occurrence of SIRS or sepsis (community-, hospital- or ICU-acquired).

Routine measurements of plasma levels of C-reactive protein (CRP) and procalcitonin (PCT) were performed according to the manufacturer’s instructions (Lumitest®; Brahms Diagnostica, Berlin, Germany).

Two blood samples (4.5 mL each) were collected from each patient. The first whole blood extraction (the DNA samples were kept at −80°C until analyzed). From the second sample the plasma was separated from leukocytes and red blood cells using collection tubes containing gel and dipotassium EDTA following centrifugation (1500 g for 10 minutes at 4°C). The plasma was stored at −80°C until analyzed. Blood collection was performed following the first episode that met SIRS criteria, either at admission or later on during the ICU stay (e.g. upon suspicion of surperinfection).

### Bacterial and Fungal DNA Detection

The bacterial and Fungal DNA detection was performed using the Multiplex PCR pathogen detection system VYOO® (SIRS-Lab GmbH, Jena, Germany). VYOO® combines culture-independent pathogen-derived nucleic acid concentration and multiplex PCR-based species detection. The multiplex PCR delivers results within 8 hours and detects 34 bacterial and 6 fungal species that cause life-threatening infections (Gram positive bacteria: *Streptococcus pneumoniae, S. pyogenes, S. sanguinis, S. agalactiae S. dysgalactiae S. mutans, Enterococcus faecium, E. faecalis, Clostridium perfringens, Staphylococcus aureus, S. epidermidis, S. saprophyticus, S. haemolyticus, S. hominis*; Gram-negative bacteria: *Escherichia coli, Pseudomonas aeruginosa, Klebsiella pneumoniae, K. oxytoca, Enterobacter cloacae, E. aerogenes, Neisseria meningitidis, Morganella morganii, Proteus mirabilis, Acinetobacter baumannii, Bacteroides fragilis, Serratia marcescens, Burkholderia cepacia, Stenotrophomonas maltophilia, Prevotella buccae, P. intermedia, P.melaninogenica*; Fungi : *Aspergillus fumigatus*, *Candida albicans C. dubliniensis, C. glabrata, C. tropicalis, C. krusei, C. parapsilosis*) as well as the five most common resistance markers (methicillin *mecA*, vancomycin *vanA*, vancomycin *vanB*, β-lactamase *blaSHV,* β-lactamase *blaCTX-M*). In order to ensure the highest clinical validity, the primer selection for the species and antibiotic resistance markers were based on the results of international studies of septic infections [47].

The pathogen cells present in 5 ml of whole blood were disrupted mechanically using glass beads (0.1/2.5 mm in diameter) and a lysis device (e. g. FastPrep®-24, MP Biomedicals, Solon, OH, USA). After a proteolytic digestion step, total DNA was isolated using a short spin column protocol. The total DNA was dissolved in an appropriate buffer provided by the manufacturer and applied to the LOOXSTER® spin column. LOOXSTER® specifically concentrates the minute quantity of bacterial and fungal DNA from the predominant human DNA. Total DNA was applied to an affinity chromatography column with a matrix-immobilized DNA binding protein that recognizes motifs within the pathogen DNA. More than 90% of the human DNA background was removed. This effect substantially increases the sensitivity of the downstream multiplex PCR protocol and simultaneously reduces the time-to-result. The concentrated pathogen DNA was directly used for multiplex PCR. The pathogen-specific amplicons generated by the PCR step were visualized by gel electrophoresis and compared with VYOO®-specific DNA length markers to identify the pathogens or resistance markers present. An alternative amplicon attribution was achieved by hybridization (AT^®^ system, Clondiag), which ensures a higher degree of specificity and sensitivity according to the manufacturer recommendations.

### Cytokine Detection

Dosage assays of plasma cytokines (IL-6, IL-8, IL-10 IL-1 Ra, MCP-1, and TNF? were performed by Bio-Plex Multiplex Cytokine Assay (BioRad Lab., Hercules, CA).

### Peptidoglycan Detection

Peptidoglycan presence was measured using the Silkworm Larvae Plasma (SLP) Reagent set Kit (Wako Pure Chemical Industries Co. Ltd., Osaka, Japan). This technique that detects peptidoglycan from both Gram-positive and Gram-negative bacteria has already been shown to be effective for peptidoglycan detection in plasma from patients with sepsis [48]. Of note, none of these results were taken into account for treating our patients since the technique was not yet validated.

### Statistical Analysis

Data were expressed as medians with the interquartile range. Numerical variables were analyzed using a nonparametric analysis of variance (Kruskal-Wallis test) followed by a Mann-Whitney *U* test for comparisons between two groups. Categorical variables were compared by the chi-square or Fisher’s exact test. Relations between two continuous variables were analyzed using the Spearman’s rank correlation test (Stata Inc., College Station, TX.).
